# Distribution and Time Trends of Refractive Errors in Japanese Spectacle Wearers

**DOI:** 10.1167/iovs.67.10.5

**Published:** 2026-08-04

**Authors:** Ryo Kawasaki, Hidehito Matsuoka, Kiyotaka Hori, Shizuka Koh, Kohji Nishida

**Affiliations:** 1 Public Health, Department of Social Medicine, Graduate School of Medicine, The University of Osaka, Suita, Japan; 2 Artificial Intelligence Center for Medical Research and Application, The University of Osaka Hospital, Suita, Japan; 3 Medical Strategy Department, JINS Inc., Tokyo, Japan; 4 Department of Innovative Visual Science, Graduate School of Medicine, The University of Osaka, Suita, Japan; 5 Department of Ophthalmology, Graduate School of Medicine, The University of Osaka, Suita, Japan

**Keywords:** myopia, refractive error, large-scale data, spectacle wearer, optical shop

## Abstract

**Purpose:**

This study aimed to estimate the distribution and temporal trends of refractive errors among spectacle wearers in Japan using a nationwide database of optical chain stores.

**Methods:**

Data from 4,525,689 individuals (53.0% women and 47.0% men; age range, 6–35 years) who purchased spectacles at 434 optical chain stores across Japan between 2021 and 2023 were analyzed. For individuals aged <15 years, prescriptions issued by ophthalmologists were used. The spherical equivalent (SE) was calculated, and its distribution was described according to age, sex, and geographic region. The annual progression rate of refractive error was estimated for individuals with multiple purchases. Linear mixed models and generalized additive mixed models were applied for the analyses.

**Results:**

The SE shifted in the myopic direction with advancing age and stabilized at approximately 30 years in men and 29 years in women (median SEs, −3.3 diopter [D] and −3.5 D, respectively). Hyperopia accounted for approximately 30% of cases at the age of 6 years but decreased significantly by the early teenage years. High myopia (SE ≤ −6 D) was observed in 1.3% of children aged <10 years. The most rapid myopic progression occurred at the age of 7 years (median, −1.1 D/year). Joinpoint analysis indicated that myopia progression decelerated between 9 and 19 years, with an additional inflection point at 12 years in women.

**Conclusions:**

Myopia progression becomes significant at the age of 6 to 7 years, highlighting the need for early surveillance and timely intervention to limit progression.

Myopia is highly prevalent in East Asia, including Japan. Its increasing global incidence led to the introduction of the term “myopia boom” in 2015 to raise awareness.^1^ This trend extends beyond Asia; a recent population-based study in the United States demonstrated a consistent increase in the prevalence rates of both myopia and high myopia over the past 50 years.^2^ The global myopic population was estimated at 2.6 billion in 2020 and is projected to reach 4.8 billion by 2050, affecting approximately one in two people worldwide.^3^

Refractive error, particularly myopia, is a major cause of avoidable visual impairment that can be effectively corrected with appropriate spectacles or contact lenses.^4^ However, severe myopia is associated with an increased risk of irreversible visual impairment, including glaucoma and retinal detachment. This finding highlights the importance of controlling myopia progression—an area in which intervention strategies have recently advanced.^5^ New treatment modalities are emerging to slow myopia progression.^6^ However, understanding when and how progression peaks remain important for designing effective intervention strategies.

Characterizing the descriptive epidemiology of myopia at a large scale, particularly through nationwide or regional studies, remains challenging. Notable large-scale cohort studies overseas include the work by Tideman et al.^7^ Analyses of population-based cohorts such as the Rotterdam Study and the Generation R Study in the Netherlands (*n* = 9891) and the Avon Longitudinal Study of Parents and Children in the United Kingdom (*n* = 2495) have suggested that accelerated axial elongation beyond the normal range of ocular growth in childhood is associated with an increased risk of subsequent myopia onset and progression. Similarly, a meta-analysis by Williams et al.^8^ of 61,946 individuals from multiple European studies demonstrated that myopia represents the greatest burden of refractive error in Europe. More recently, real-world data have been used to track refractive error progression trajectories. The European DREAM Study [Drentse Refractive Error and Myopia] used spectacle prescription data from optical shops to monitor myopia progression from childhood to adulthood.^9^ Moreover, large-scale medical record databases have also been used to longitudinally track refractive changes.^2^

Myopia is a major public health concern in Japan. A 2022 survey of 8846 elementary and junior high school students by the Ministry of Education, Culture, Sports, Science, and Technology reported that approximately 25% of first-grade elementary school students and 60% of third-year junior high school students had uncorrected visual acuity below 1.0.^10^ Furthermore, Takeuchi et al.^11^ conducted a large-scale case-series study involving 593,273 patients at a single hospital in Yokohama. Although studies based on school populations or single institutions have been conducted, comprehensive nationwide or region-wide descriptive data remain limited. Consequently, the distribution and temporal trends of refractive errors across the spectrum from childhood to adulthood in Japan have not yet been fully elucidated. Recent consensus emphasizes that clinically meaningful myopia progression initiated at a younger age may continue into young adulthood; moreover, delaying the onset of myopia by even 1 year may confer substantial lifelong benefits.^12^ As monofocal lenses remain the most commonly prescribed optical correction worldwide,^13^^,^^14^ spectacle lens prescriptions were hypothesized to serve as an indicator of the distribution and trends of refractive errors, particularly when population coverage is sufficiently large.

Therefore, this study aimed to estimate the distribution of refractive errors across the full spectrum, including emmetropia, myopia, and hyperopia, using optical prescriptions obtained from a nationwide optical chain store database derived from customer records of a large nationwide optical shop group. This approach was used to characterize the real-world distribution and temporal trends of myopia in Japan and to estimate the distribution of myopic refractive error according to age, sex, and region and the progression rate of refractive errors toward the myopic direction.

## Methods

### Study Design, Participants, and Data Source

This observational study incorporated both cross-sectional and longitudinal components. The study was approved by the Institutional Review Board of Osaka University Hospital (approval number: 24231(T1)) and conducted using an opt-out consent approach. All data were completely anonymized before analysis.

The data source comprised spectacle prescription records from a nationwide optical chain (JINS Co., Ltd., Maebashi, Japan), which holds approximately 33% of the market share and covers approximately 9,000,000 spectacle wearers. Baseline demographic variables included age, sex, date of purchase, and store location. Refractive information included spherical and cylindrical powers, from which the spherical equivalent (SE) was calculated as follows: SE (diopter, D) = spherical power (D) + (0.5 × cylinder power [D]).

In Japan, the School Health Law mandates annual uncorrected distance visual acuity examinations for all students. After screening, ophthalmological prescriptions are provided for individuals with uncorrected decimal visual acuity below 1.0, to achieve a target distance visual acuity of ≥1.0. For individuals aged <15 years, spectacle prescriptions were based on those issued by ophthalmologists. All lenses prescribed during the study period were conventional single-vision lenses, because myopia control lenses were not yet available.

Two cohorts were defined using this data source. For the cross-sectional analysis, all unique purchase records from September 1, 2021, to August 31, 2023, for individuals aged 6 to 35 years were included. For the longitudinal analysis, repeat purchase records were used to estimate myopia progression rates.

### Myopia Classification

The proportion of refractive errors at each age was calculated by classifying individuals into five categories: hyperopia (≥+0.50 D), emmetropia (−0.49 D to +0.49 D), mild myopia (−0.50 D to −2.99 D), moderate myopia (−3.00 D to −5.99 D), and high myopia (≤−6.0 D).^1^

### Regional Comparison

Nine standard geographic regions were defined: Hokkaido, Tohoku, Kanto, Chubu, Kansai, Chugoku, Shikoku, Kyushu, and Okinawa. The median SE values for each region and age were compared using spline curves. The Kanto region was selected as the reference region, because it had the highest number of purchases.

### Cross-Sectional Analysis

Sex differences in SE across age were evaluated using box plots stratified by age and sex. A mixed-effects model was applied to estimate individual representative SE values while accounting for intereye correlation. Generalized additive mixed models (GAMMs) were used to compare age-related refractive changes by sex and region. In the GAMMs, the SE for each eye was the dependent variable, and age and sex or region were the independent variables. Smoothing spline functions modeled nonlinear effects of age and region, and interaction smooth terms were added to evaluate differences in age-related effects by sex or region. To account for intereye correlation, a unique participant ID was incorporated as a random effect using a random effect spline term. Models were estimated using restricted maximum likelihood. Population-averaged predicted values adjusted for intereye correlation were calculated by sex or region and visualized as smoothed age-related curves. Joinpoint regression analysis using the weighted Bayesian information criterion identified ages at which significant changes in the SE occurred.

### Longitudinal Analysis to Determine the Progression Rate

Myopia progression rates were calculated and summarized by sex and age at initial purchase. To avoid overestimating progression, all eligible participants were included, including those with no refractive change (0.00 D/year) or a shift toward hyperopia (positive annualized SE change). The median myopia progression rate was also estimated for subgroups defined by age at initial purchase and baseline SE. A mixed-effects model was applied to estimate progression rates according to baseline SE while accounting for intereye correlation. GAMMs were used to compare age-related changes in progression rates between sexes. The progression rate for each eye was the dependent variable, with age and sex included as independent variables. Smoothing spline functions modeled nonlinear age effects, and interaction smooth terms were added to evaluate differences in age-related effects by sex. To adjust for the correlation between the measurements of the left and right eyes, the unique participant ID assigned to each individual was incorporated as a random effect using a random-effect spline term. Models were estimated using restricted maximum likelihood. Population-averaged predicted values adjusted for intraindividual intereye correlation were calculated by sex and visualized as smoothed age-related curves. Finally, a Joinpoint regression analysis was then performed to identify significant changes in age-related progression trends.

Data aggregation and visualization were conducted using Python (version 3.12). GAMM analyses were performed using the *mgcv* package in R (version 4.4), and Joinpoint regression was conducted using Joinpoint Trend Analysis Software (version 6.0; National Cancer Institute, Bethesda, MD). Multiple joinpoints were permitted, and permutation tests were used to determine the optimal number. Final model selection was based on the weighted Bayesian information criterion. Significance was set at a two-sided *P* value of <0.05.

## Results

### Baseline Characteristics in the Cross-Sectional Analysis

**Figure 1.**
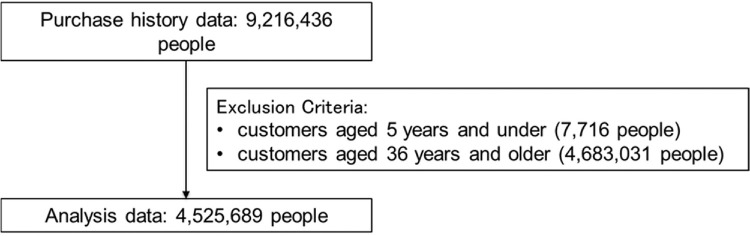
Flowchart of study participants included in the cross-sectional analysis. The initial dataset comprised 9,216,436 participants with purchase history records. After excluding individuals aged ≤5 years (*n* = 7716) and those aged ≥36 years (*n* = 4,683,031), 4,525,689 participants were included in the final cross-sectional analysis.

**Figure 2.**
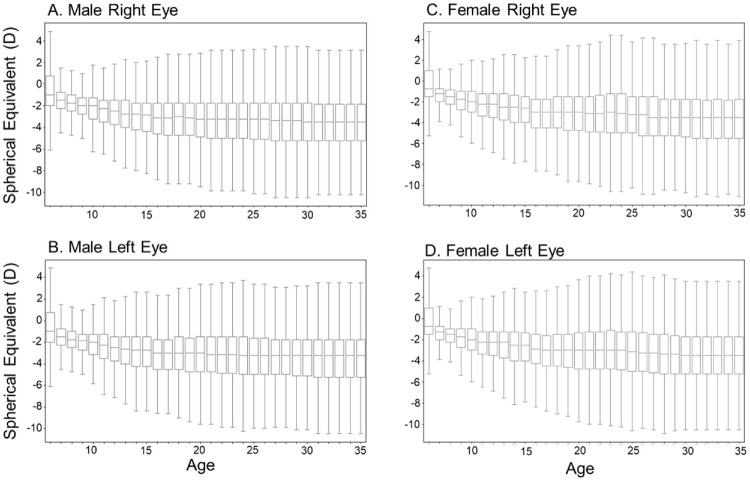
Age-related distribution of the SE. Box plots illustrate the distribution of the SE (D) across ages 6 to 35 years. Data are stratified by sex and eye: (**A**) male right eye, (**B**) male left eye, (**C**) female right eye, and (**D**) female left eye.

Unique purchase records from 9,216,436 individuals (September 1, 2021, to August 31, 2023) were initially included in the cross-sectional analysis. After excluding individuals outside the 6- to 35-year age range, 4,525,689 participants remained for analysis (Fig. 1), of whom 53.0% were women (mean age, 22.4 years). Mean SE values were −3.3 D in the right eye and −3.2 D in the left eye. Detailed characteristics are presented in Table 1. A clear age-dependent decline in SE was observed during early life in both sexes, reflecting myopia progression. In men, the median SE decreased from −1.0 D (both eyes) at age 6 years to −3.5 D (right eye) and −3.25 D (left eye) by age 30 years. Among women, the median SE declined from −0.75 D (both eyes) at age 6 years to −3.5 D (both eyes) by age 30 years. Thereafter, SE remained largely stable (Fig. 2, Supplementary Table S1). Sex-based differences in SE trajectories were significant according to GAMM analysis (*P* < 0.001). In younger age groups, men tended to exhibit stronger myopia than women; however, after approximately 26 years of age, women exhibited higher degrees of myopia (Supplementary Fig. S1). In both sexes, the rate of myopia progression appeared to slow from the early to late 20s.

**Table 1.** Baseline Characteristics of Participants Included in the Cross-Sectional Analysis

Characteristics	Values
Total number of people	4,525,689
Sex	
Female	2,397,025 (53.0)
Male	2,128,664 (47.0)
Age, years	22.4 ± 7.5
Age, years	
6–9	165,266 (3.7)
10–19	1,481,598 (32.7)
20–29	1,904,214 (42.1)
30–35	974,611 (21.5)
Region	
Hokkaido	152,553 (3.4)
Tohoku	301,808 (6.7)
Kanto	1,899,342 (42.0)
Chubu	689,632 (15.2)
Kansai	750,685 (16.6)
Chugoku	230,651 (5.1)
Shikoku	108,589 (2.4)
Kyushu	348,557 (7.7)
Okinawa	43,872 (1.0)
SE (right)	−3.3 ± 2.3
SE (left)	−3.2 ± 2.3

Values are number (%) for categorical variables or mean ± standard deviation for continuous variables. D = diopters. The total analysis population included individuals aged 6 to 35 years.

**Figure 3.**
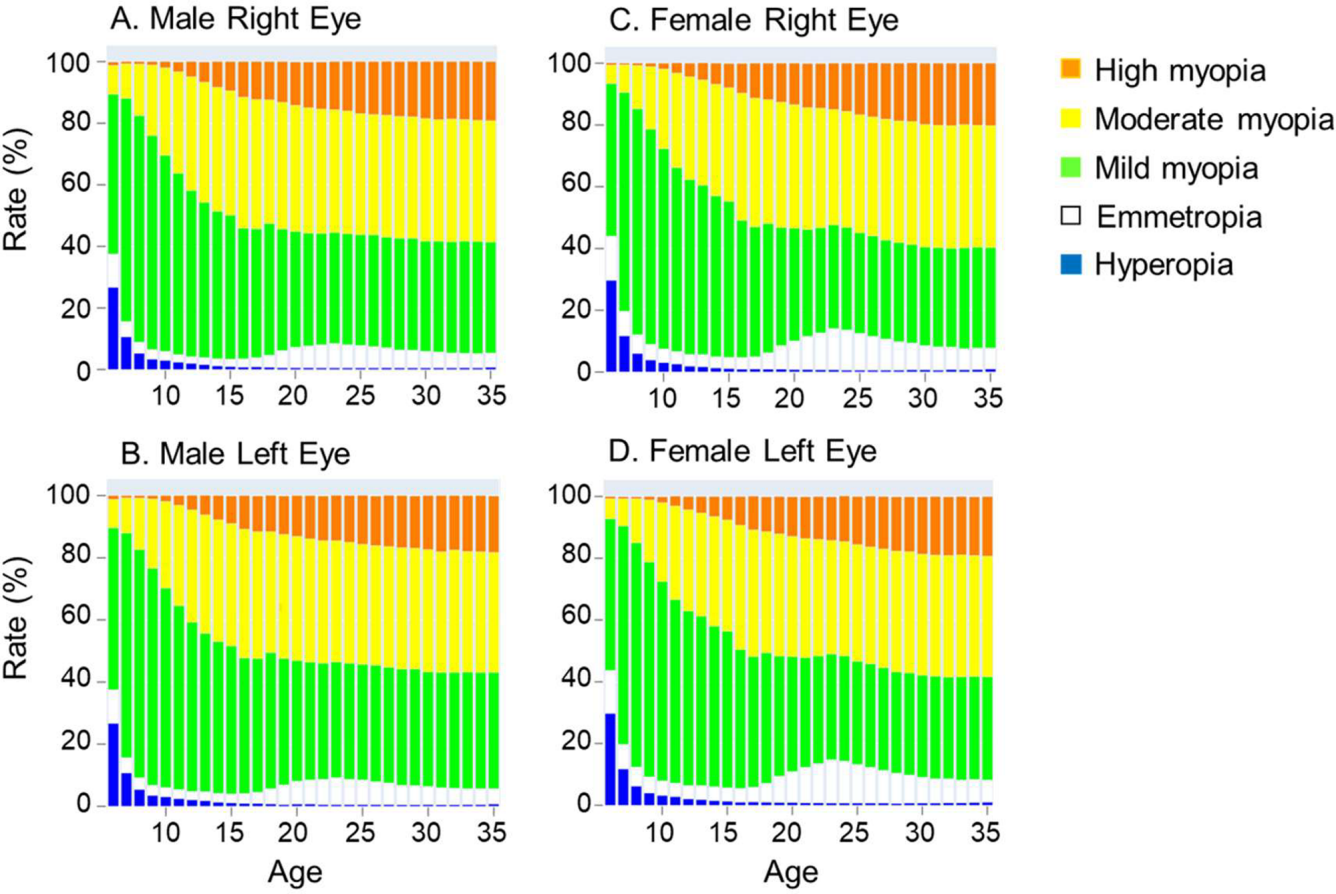
Distribution of refractive error categories by age. Stacked bar charts demonstrate the proportion (%) of patients with hyperopia (≥+0.50 D), emmetropia (−0.49 D to +0.49 D), mild myopia (−0.50 to −2.99 D), moderate myopia (−3.00 to −5.99 D), and high myopia (SE ≤−6.0 D) according to age. (**A**) Male right eye, (**B**) male left eye, (**C**) female right eye, and (**D**) female left eye.

Analysis of myopia severity revealed that SE decreased with age and stabilized after age 30 years in men and 29 years in women, according to Joinpoint analysis (Supplementary Fig. S2). Conversely, the proportion of individuals with high myopia increased from approximately 10 years of age in both sexes. Notably, high myopia was observed in 1.3% of individuals aged <10 years (Fig. 3). Hyperopia accounted for approximately 30% at age 6 years; however, this proportion decreased rapidly with age, becoming negligible by early adolescence.

Regional analysis of age-related SE changes demonstrated a similar pattern of myopia progression across the nine regions of Japan (Supplementary Figs. S3A–S3C). However, when the median SE values across all ages were compared, GAMM analysis revealed that the median SE is higher in the Tohoku region, and lower in the Hokkaido and Okinawa regions than in the Kanto region (*P* < 0.01, *P* < 0.05, and *P* < 0.05) in men. In Okinawa, the SE shifted in a more myopic direction among individuals aged 15 to 20 years but in a less myopic direction among those aged 25 to 30 years.

### Myopia Progression Rate in the Longitudinal Analysis

**Figure 4.**
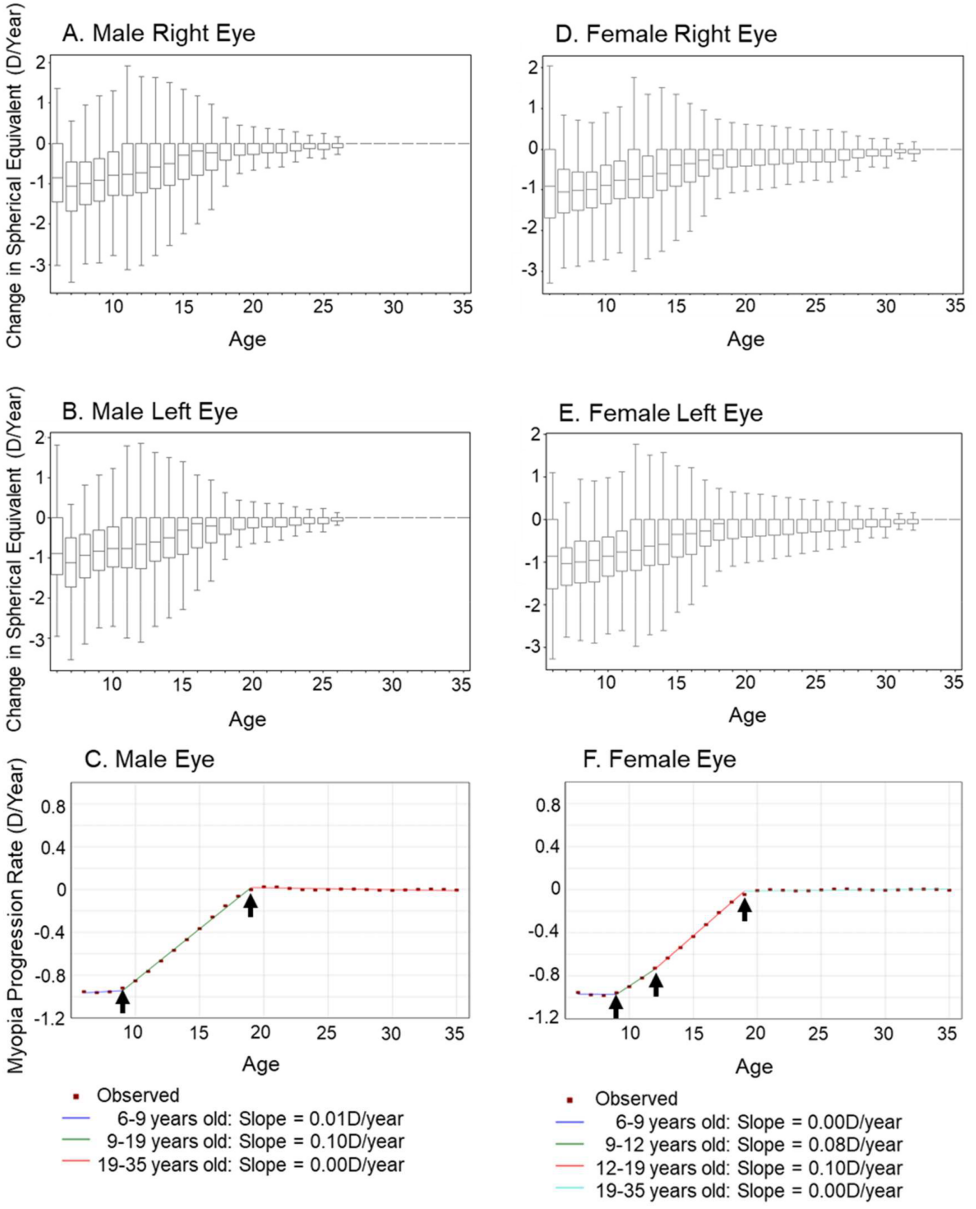
Age-specific myopia progression rates and joinpoint analysis. Box plots show changes in the SE (D/year) by age at the initial visit for men (**A**, **B**) and women (**D**, **E**). (**C**, **F**) Joinpoint regression analysis of myopia progression rates (D/year) for men and women, respectively; asterisks indicate slopes significantly different from zero at the alpha = 0.05 level.

**Figure 5.**
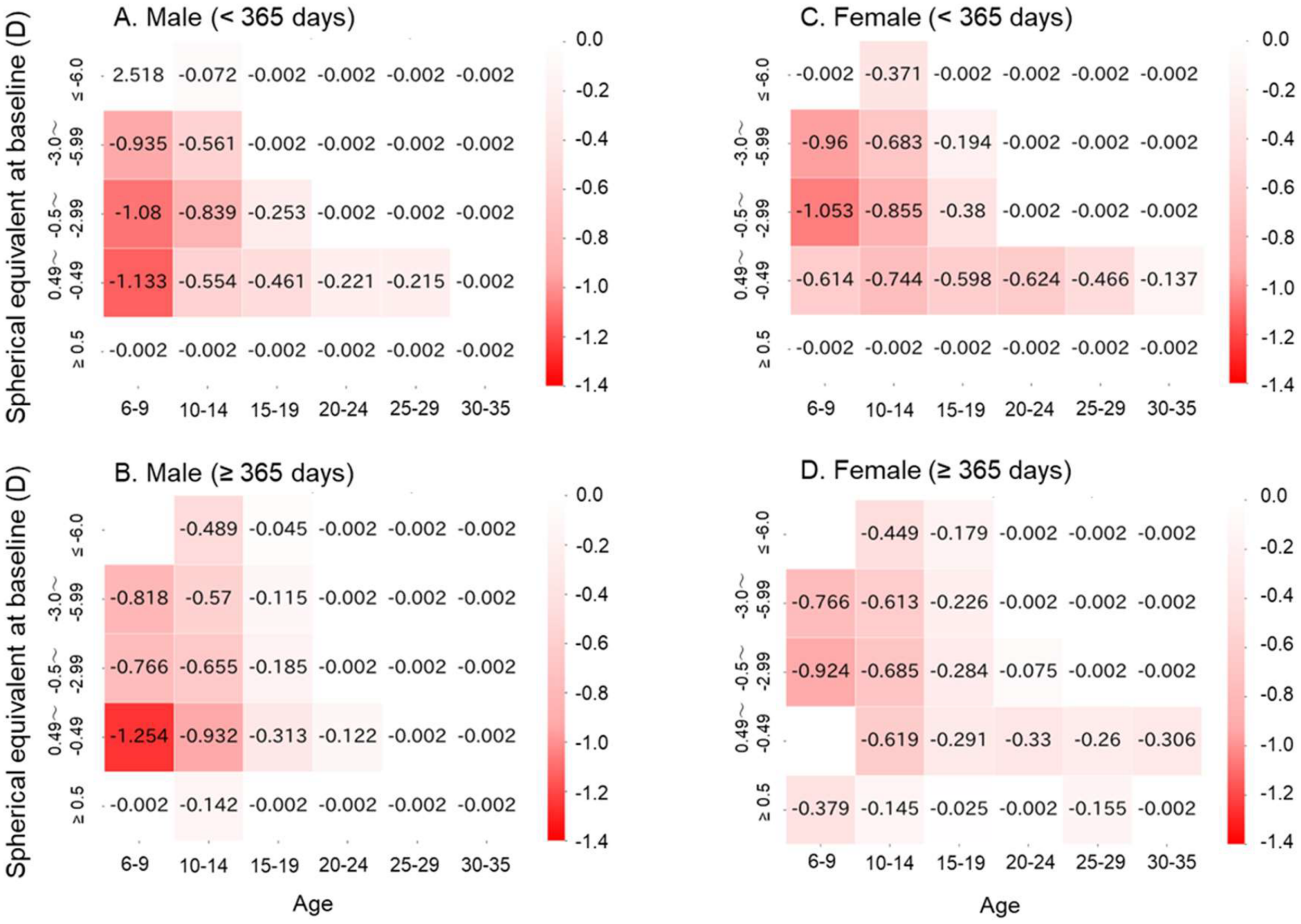
Heatmaps of annual refractive progression rates by baseline SE and age group. Annual progression rates (D/year) were estimated using mixed-effects models. Data are stratified by baseline age group, baseline SE category, and follow-up interval. (**A**, **C**) Follow-up intervals of <365 days for men and women, respectively. (**B**, **D**) Follow-up intervals of ≥365 days for men and women, respectively.

Multiple purchase records were used to estimate myopia progression rates. Participants with repeated purchases were identified, and after a multistage quality filtering based on the predefined exclusion criteria, 133,483 individuals were included in the longitudinal cohort (Supplementary Fig. S4; Table 2). Analysis of the interval between the first and second spectacle purchases showed that progression was fastest at the age of 7 years in both sexes. Median progression rates were −1.1 D/year in both eyes for men and −1.1 D/year (right eye) and −1.0 D/year (left eye) for women (Figs. 4A, 4B, 4D, 4E; Supplementary Table S2). A Joinpoint regression analysis identified the ages at which progression trends changed significantly. Two joinpoints were detected in men (ages 9 and 19 years) and three in women (ages 9, 12, and 19 years) (Figs. 4C and 4F). Annual progression rates estimated using mixed-effects models were visualized as heatmaps (Fig. 5). Progression was most pronounced in the 6- to 9- and 10- to 14-year age groups across all baseline SE categories. Notably, men aged 6 to 9 years with a baseline SE of +0.49 D to −0.49 D (follow-up of ≥365 days) demonstrated the highest progression rate (−1.25 D/year). Progression generally decelerated to below −0.3 D/year by age 20 years. Shorter observation intervals (<365 days) occasionally showed higher progression rates, possibly reflecting sampling bias among individuals with rapid refractive changes who returned earlier (Fig. 5).

**Table 2.** Baseline Characteristics of Participants Included in the Longitudinal Analysis

Characteristics	Value
Total number of people	133,483
Sex	
Female	64,816 (48.6)
Male	68,667 (51.4)
Age, years	24.9 ± 6.7
Age, years	
6–9	2127 (1.6)
10–19	23,794 (17.8)
20–29	71,174 (53.3)
30–35	36,388 (27.3)
Region	
Hokkaido	4927 (3.7)
Tohoku	9124 (6.8)
Kanto	58,086 (43.5)
Chubu	20,701 (15.5)
Kansai	19,746 (14.8)
Chugoku	6843 (5.1)
Shikoku	3309 (2.5)
Kyushu	9477 (7.1)
Okinawa	1270 (1.0)
SE (right)	−3.5 ± 2.3
SE (left)	−3.4 ± 2.3

Values are number (%) for categorical variables or mean ± standard deviation for continuous variables. Baseline characteristics were recorded at the time of the initial visit for individuals with multiple purchases.

## Discussion

This study characterized the descriptive epidemiological profile of refractive errors and myopia among spectacle users in Japan at an unprecedented scale, covering one-third of national prescriptions. Although it does not represent true prevalence, longitudinal findings demonstrated that myopia progresses from age 6 years to 30 years in men and up to 29 years in women, with stabilization thereafter. This delayed stabilization of myopia observed in this study provides a novelty from real-world data of prescribed spectacles. Although previous longitudinal studies (e.g., COMET [Correction of Myopia Evaluation Trial]^15^ and DREAM^9^) reported stabilization between 18 and 21 years of age, our large real-world database demonstrates that clinically meaningful myopic shifts continue throughout the 20s. This finding aligns with recent International Myopia Institute insights highlighting common adult progression between 20 and 30 years.^16^ These findings may inform future research and the clinical management of myopia in young adults.

Key findings for future research and intervention include early myopia onset at 6 to 7 years of age, highlighting the importance of early monitoring and intervention. Sex-related differences in myopia progression were also observed. High myopia was present in 1.3% of children aged <10 years, whereas hyperopia (approximately 30% at age 6 years) decreased to negligible levels by early adolescence. The extended progression period up to approximately 30 years provides context for the high prevalence observed in older adults. For example, the Nagahama Study reported that among Japanese adults aged 35 to 59 years, the prevalence of myopia and high myopia reached approximately 70% and 10%, respectively.^17^ Collectively, these findings highlight a continuous and marked myopic shift from childhood into adulthood in Japan.

In East Asia, including Japan, the prevalence of myopia among school-aged children has markedly increased in recent years, becoming a major public health concern.^18^^,^^19^ Myopia onset and progression are thought to be influenced by environmental factors, behavioral patterns, and genetic predisposition, including parental myopia.^20^ The observed female predominance in young adults may reflect a recent shift driven by environmental and lifestyle factors, and is also supported by faster progression in girls during childhood.^21^^,^^22^ Regional variations observed in this study may partly reflect variation in environmental and behavioral determinants of myopia, particularly sunlight exposure and outdoor activity. Jones et al.^23^ reported that children with one or both parents with myopia had a lower risk of myopia when engaging in greater levels of sports and outdoor activities. Similarly, Rose et al.^24^ demonstrated that children in Sydney, Australia, who spent more time outdoors had a lower risk of myopia onset. The finding that men in the Hokkaido and Okinawa regions differed significantly and nonlinearly from those in Kanto region is noteworthy, given the distinct geographical characteristics of these areas (Supplementary Fig. S3). Higher myopia prevalence in certain regions may partly reflect environmental differences. Northern regions such as Hokkaido are characterized by heavier snowfall, higher precipitation, and greater cloud cover, resulting in reduced sunlight exposure, which has been associated with an increased myopia risk. (Data from the Japan Meteorological Agency [https://www.data.jma.go.jp/stats/data/mdrr/tenkou/indexTenkou.html].) However, our findings of more myopic SE in those 15 to 20 years old and less myopic SE in those 25 to 30 years old in Okinawa suggest a complex age-dependent pattern, more specifically a cohort effect related to generational lifestyle shifts. Further analyses such as age–period–cohort modeling are needed to clarify this finding. The study period (2021–2023), which coincided with the COVID-19 pandemic, should be considered. During this time, public health measures led to substantial lifestyle changes, including reduced outdoor activity and increased screen time.^25^ Although we do not have enough data to determine how COVID-19 influenced the trends of myopia in Japan, these environmental shifts have been hypothesized to accelerate the onset and progression of myopia globally and may have influenced the trends observed in this cohort. Accordingly, long-term data monitoring is important to clarify the potential impact of COVID-19 on myopia, a phenomenon sometimes referred to as quarantine myopia.^26^^,^^27^

In this study, 1.3% of children aged <10 years who had already purchased spectacles were classified as having high myopia. A Tokyo metropolitan survey reported higher prevalence rates of high myopia (SE ≤−6.0 D) of 4.0% in elementary school students and 15.2% in junior high school students.^28^ Because reducing high myopia is a key goal of myopia control, these findings underscore the importance of early monitoring for high myopia in this population. However, interpretation should consider a nationwide claims database study reporting a much lower prevalence of 0.46% in individuals aged 10 to 14 years.^29^ The higher proportion in this cohort likely reflects selection bias in a spectacle-seeking population.^29^

Regarding myopia progression, Takeuchi et al.^11^ reported 5-year longitudinal refractive changes in a single-hospital cohort in Japan. Both that study^11^ and ours demonstrated active pediatric myopia progression, particularly in early school years. The peak progression age in this study (6–7 years) was broadly consistent with their reported peak at 8 years, underscoring the importance of early-life myopia prevention. However, progression rates differed: the hospital-based cohort reported approximately −0.5 D/year in boys and −0.6 D/year in girls at ages 6 to 8, whereas our spectacle-based cohort showed approximately −1.1 D/year in both sexes, indicating nearly two-fold higher rates in prescription-based estimates. This discrepancy may reflect differences in study populations and methods. Our optical store cohort likely over-represents individuals with rapid myopia progression due to spectacle-seeking behavior, whereas hospital-based cohorts such as that in a report by Takeuchi et al.^11^ include broader populations and yield lower averages. Methodologically, our shorter follow-up intervals (<1–2 years) captured rapid changes that may be attenuated in 5-year averages. This may also reflect Japan's School Health Law, which mandates annual screening and facilitates earlier spectacle updates.

Recent studies (e.g., STAR [STudy of Atropine to Reduce]^30^ and the European Delphi consensus^31^) define rapid myopia progressors as those with progression of ≤−0.75 D/year. In our cohort, individuals updating prescriptions within 365 days often met or exceeded this threshold (Fig. 5). These rapid myopic shifts were likely accelerated by the COVID-19 pandemic (2021–2023) due to increased screen time and decreased outdoor activities. Thus, despite a limited follow-up duration, our approach effectively identified rapid progressors during this unique period. This prescription-based monitoring approach enables the identification of rapid progressors at a large scale, a capability that is uniquely facilitated by prescription databases. Despite differences in absolute progression rates, the consistent age-related trend observed across studies suggest that optical store data provide valuable insights into myopia patterns, particularly within the spectacle-wearing population. This critical timing is further corroborated by a nationwide claims database study demonstrating that the incidence of myopia in Japan peaks at age 8 years.^29^

Comparison with a nationwide school-based survey by the Ministry of Education, Culture, Sports, Science, and Technology (ages 6–15 years) provides additional context (Supplementary Fig. S5).^32^ The national survey, based on 29 sampled elementary and junior high schools across Japan, included all students regardless of myopia severity or uncorrected distance visual acuity; it therefore provides a more accurate estimate of refractive error distribution. In contrast, this study included individuals who voluntarily purchased spectacles, encompassing children whose perceived visual impairment prompted optical correction. Accordingly, the refractive distribution in this study was approximately −1.5 D more myopic at each age compared with that reported in the ministry's survey. The pattern of relative myopia increase was similar across sexes (Supplementary Fig. S5). This difference likely reflects referral pathways from school screening and ophthalmological care. In Japanese school health examinations, uncorrected distance visual acuity is categorized as ≥1.0 (category A), 0.9 to 0.7 (category B), 0.6 to 0.3 (category C), or ≤0.2 (category D).^32^ Referral for ophthalmologic evaluation is typically recommended for categories C and D. Mean SE values for categories C and D are −1.94 D (median, −1.75 D) and −3.68 D (median, −3.49 D), respectively.^32^ The differences observed between the findings of our study and those of the ministry's survey may reflect the lack of referral for individuals in category B (0.9–0.7). Whether children in this category, with a mean SE of −0.81 D and a median SE of −0.75 D,^32^ require spectacle correction warrants further consideration.

Peak myopia progression in this study occurred at 6 to 9 years of age in both sexes (Fig. 5, Supplementary Fig. S2). Because progression rates may be higher in individuals with greater baseline myopia, the estimates reported here are likely higher than those of the general population, which includes individuals without refractive error. Furthermore, the observed progression rates, together with evidence of earlier myopia onset—particularly the rapid progression at 6 to 7 years of age—highlight the need for targeted interventions during this critical period. Because myopia-control spectacle lenses were not commercially available in Japan until June 2026, all children in our 2021–2023 cohort exclusively used conventional monofocal lenses. Thus, our rapid progression rates reflect the natural trajectory of myopia without confounding optical interventions, highlighting the urgent need for effective myopia control strategies in Japan.

This study demonstrated that data obtained from optical stores provide several insights into the distribution and trends of refractive errors in real-world spectacle prescriptions. First, because such data are continuously accumulated, they enable analyses at any desired time point and age range, provided that repeated spectacle purchases are available. Second, linkage via customer identifiers, with appropriate consent, allows the potential collection of supplementary background information—such as lifestyle habits and socioeconomic factors—through questionnaires. These features support the development of longitudinal studies capable of tracking temporal changes in refractive status. In the future, large-scale accumulated datasets may enable the application of advanced predictive modeling and pattern recognition approaches, including those based on artificial intelligence.

This study has some limitations. First, the data were obtained from spectacle purchasers, likely resulting in a cohort with a higher distribution and severity of symptomatic myopia than the general population. Consequently, the estimates of myopia distribution and progression rates may not fully reflect population-level patterns and are likely biased toward more myopic refractive values. Second, despite its large scale, the dataset was not exhaustive and may not fully represent the entire Japanese population. This limitation may be further influenced by regional variations; areas with fewer samples or a lower density of store locations may yield less reliable estimates. Furthermore, the low percentage of children and adolescents limits our findings on myopia progression, because the greatest progression is observed in this age group. Third, this 2-year observation period used to calculate the progression rates may have preferentially captured individuals with faster myopia progression, because those with slower progression may not have required a new prescription within this timeframe. Therefore, long-term data accumulation will enable more detailed analyses. Finally, the analysis was based on spectacle prescription data rather than direct clinical measurements; thus, the calculated SE may not fully reflect true physiological refractive error. Furthermore, as secondary data, the dataset lacked detailed information on previous refractive surgery, pseudophakia, or cataract status. The use of cycloplegia during clinical examinations could also not be verified. Moreover, no information regarding lifestyle factors—such as outdoor activity—was available, limiting comprehensive interpretation of the results.

## Conclusions

This study analyzed real-world spectacle prescription data covering approximately one-third of the Japanese optical market and described the epidemiological distribution and patterns of refractive errors. Notably, myopia progression extended into young adulthood, with stabilization occurring later than typically reported (approximately 30 years in men and 29 years in women). Furthermore, our longitudinal approach captured rapid refractive changes within <1 year, underscoring the need for frequent monitoring and early intervention. Together with observed sex and regional differences, these findings provide insight into real-world refractive management. This study highlights the potential of optical prescription data as a valuable resource for further refractive error research, particularly for longitudinal analyses using repeated prescriptions.

## Supplementary Material

Supplement 1
