# Supplementary material for: Distribution and Time Trends of Refractive Errors in Japanese Spectacle Wearers
Source: Invest Ophthalmol Vis Sci. 2026 Aug 4;67(10):5. doi: 10.1167/iovs.67.10.5 (PMC13446541; doi:10.1167/iovs.67.10.5)
Supplement: Supplementary file 1 [file iovs-67-10-5_s001.pdf]

Supplementary Materials

Supplementary Table S1. Median and interquartile range of spherical equivalent by age, sex, and eye.

Male											
Age	n	Right Eye					Left Eye				
		Mid.	25%	75%	Min.	Max.	Mid.	25%	75%	Min.	Max.
6	5,234	−1.00	−2.00	0.75	−16.75	8.75	−1.00	−2.00	0.75	−13.88	9.00
7	13,299	−1.50	−2.25	−0.75	−13.25	9.63	−1.50	−2.25	−0.75	−13.75	10.38
8	25,001	−1.75	−2.50	−1.00	−14.88	9.50	−1.75	−2.50	−1.00	−13.88	8.50
9	35,871	−2.00	−2.75	−1.25	−13.00	9.50	−1.88	−2.75	−1.25	−11.00	9.50
10	44,148	−2.00	−3.25	−1.25	−16.75	9.50	−2.00	−3.13	−1.25	−17.63	8.50
11	51,670	−2.25	−3.50	−1.50	−17.00	9.00	−2.25	−3.50	−1.25	−14.75	9.00
12	68,973	−2.50	−3.75	−1.50	−14.50	10.50	−2.50	−3.75	−1.50	−14.50	10.75
13	67,605	−2.75	−4.00	−1.50	−17.50	9.00	−2.63	−4.00	−1.50	−17.00	8.75
14	72,826	−2.75	−4.25	−1.75	−16.50	9.88	−2.75	−4.25	−1.50	−17.25	9.50
15	83,398	−2.88	−4.38	−1.75	−15.50	9.63	−2.75	−4.25	−1.50	−16.25	10.38
16	62,441	−3.13	−4.63	−1.75	−16.00	8.50	−3.00	−4.50	−1.75	−15.50	9.00
17	70,342	−3.13	−4.75	−1.75	−16.00	8.50	−3.00	−4.50	−1.75	−16.50	8.13
18	99,348	−3.00	−4.75	−1.75	−15.00	7.13	−3.00	−4.50	−1.50	−16.00	8.75
19	77,719	−3.13	−4.75	−1.75	−16.25	7.50	−3.00	−4.75	−1.63	−17.25	7.75
20	79,720	−3.25	−4.88	−1.75	−17.00	8.00	−3.00	−4.75	−1.50	−17.63	8.63
21	80,734	−3.25	−5.00	−1.75	−16.88	8.50	−3.13	−4.75	−1.50	−15.50	9.00
22	88,436	−3.25	−5.00	−1.75	−18.00	12.13	−3.13	−4.88	−1.50	−18.00	11.00
23	87,414	−3.25	−5.00	−1.75	−16.00	8.00	−3.13	−4.88	−1.50	−15.75	9.00
24	89,790	−3.25	−5.00	−1.75	−18.00	7.88	−3.25	−5.00	−1.50	−18.00	9.13
25	88,778	−3.25	−5.13	−1.75	−17.00	9.13	−3.25	−5.00	−1.63	−16.50	9.13
26	86,875	−3.25	−5.13	−1.75	−17.63	10.00	−3.25	−5.00	−1.63	−16.63	9.00
27	87,944	−3.38	−5.25	−1.75	−16.63	8.50	−3.25	−5.00	−1.75	−16.13	9.13
28	88,150	−3.38	−5.25	−1.75	−17.50	8.75	−3.25	−5.00	−1.75	−18.00	9.00
29	86,093	−3.38	−5.25	−1.75	−18.00	10.50	−3.25	−5.13	−1.75	−15.75	7.75
30	85,474	−3.50	−5.25	−1.75	−18.00	10.50	−3.25	−5.13	−1.75	−18.00	10.50
31	82,096	−3.50	−5.25	−1.88	−17.88	8.50	−3.25	−5.25	−1.75	−17.25	8.75
32	80,742	−3.50	−5.25	−1.88	−18.00	10.00	−3.25	−5.25	−1.75	−18.00	10.00
33	79,244	−3.50	−5.25	−1.88	−17.38	12.50	−3.25	−5.25	−1.75	−16.88	12.13
34	79,244	−3.50	−5.25	−1.88	−17.75	9.25	−3.25	−5.25	−1.75	−18.00	9.75
35	80,055	−3.50	−5.25	−1.88	−17.25	9.25	−3.25	−5.25	−1.75	−18.00	9.25

Female											
Age	n	Right Eye					Left Eye				
		Mid.	25%	75%	Min.	Max.	Mid.	25%	75%	Min.	Max.
6	4,701	−0.75	−1.50	1.00	−16.00	9.13	−0.75	−1.50	1.00	−14.00	9.00
7	12,969	−1.25	−2.00	−0.75	−16.75	9.00	−1.25	−2.00	−0.75	−13.50	9.13
8	26,807	−1.50	−2.25	−0.88	−11.25	9.00	−1.50	−2.25	−1.00	−11.38	9.00
9	41,384	−1.75	−2.75	−1.00	−17.75	8.50	−1.75	−2.75	−1.00	−15.75	9.00
10	55,891	−2.00	−3.00	−1.00	−14.75	9.25	−2.00	−3.00	−1.00	−13.25	9.50
11	67,662	−2.25	−3.38	−1.25	−15.50	9.00	−2.25	−3.38	−1.25	−15.00	9.50
12	84,374	−2.25	−3.50	−1.25	−16.25	9.38	−2.25	−3.50	−1.25	−17.50	9.75
13	81,159	−2.50	−3.75	−1.25	−17.50	9.00	−2.25	−3.75	−1.25	−17.50	9.00
14	85,335	−2.50	−4.00	−1.38	−15.00	9.00	−2.50	−4.00	−1.25	−15.75	9.25
15	87,833	−2.63	−4.00	−1.50	−15.63	10.00	−2.50	−4.00	−1.38	−14.50	10.25
16	67,383	−3.00	−4.50	−1.75	−16.50	8.75	−2.88	−4.25	−1.50	−18.00	8.75
17	73,930	−3.00	−4.50	−1.75	−15.13	9.75	−3.00	−4.50	−1.63	−15.50	9.25
18	94,794	−3.00	−4.50	−1.50	−18.00	8.50	−3.00	−4.50	−1.50	−18.00	8.50
19	84,767	−3.00	−4.75	−1.50	−17.38	8.00	−3.00	−4.63	−1.50	−16.25	7.75
20	94,393	−3.00	−4.75	−1.50	−17.50	9.00	−3.00	−4.75	−1.38	−16.25	8.50
21	102,151	−3.13	−4.88	−1.50	−15.75	11.00	−3.00	−4.75	−1.25	−15.75	9.50
22	113,320	−3.13	−4.88	−1.38	−17.25	11.50	−3.00	−4.75	−1.25	−17.00	12.00
23	114,338	−3.00	−5.00	−1.25	−18.00	8.00	−3.00	−4.75	−1.13	−16.75	8.50
24	110,955	−3.13	−5.00	−1.25	−17.00	8.38	−3.00	−4.88	−1.25	−16.88	9.00
25	107,813	−3.25	−5.00	−1.50	−17.00	9.50	−3.13	−5.00	−1.25	−17.00	9.00
26	103,515	−3.25	−5.25	−1.50	−18.00	9.88	−3.25	−5.00	−1.38	−17.25	9.88
27	101,145	−3.50	−5.25	−1.50	−17.50	8.25	−3.25	−5.13	−1.50	−18.00	8.75
28	99,295	−3.50	−5.25	−1.75	−16.75	10.63	−3.38	−5.25	−1.50	−16.88	10.63
29	93,355	−3.50	−5.25	−1.75	−16.75	9.50	−3.38	−5.25	−1.63	−16.50	9.13
30	90,119	−3.50	−5.38	−1.75	−17.50	10.00	−3.50	−5.25	−1.75	−17.50	9.50
31	84,052	−3.50	−5.50	−1.75	−18.00	9.00	−3.50	−5.25	−1.75	−18.00	10.00
32	82,019	−3.50	−5.50	−1.88	−17.00	10.00	−3.50	−5.25	−1.75	−17.88	10.00
33	78,444	−3.50	−5.50	−1.75	−17.00	8.75	−3.50	−5.25	−1.75	−18.00	9.50
34	76,853	−3.50	−5.50	−1.88	−18.00	8.00	−3.50	−5.25	−1.75	−18.00	8.00
35	76,269	−3.50	−5.50	−1.75	−18.00	8.50	−3.50	−5.25	−1.75	−18.00	10.00

This table presents the precise values (median, 25th percentile, and 75th percentile) of the spherical equivalent (diopters) for each age group (6 to 35 years), stratified by sex and right/left eye, corresponding to the data visualized in Figure 2.

Supplementary Table S2. Median and interquartile range of annualized myopia progression rate by age at initial visit and sex.

Male											
Age	n	Right Eye					Left Eye				
		Mid.	25%	75%	Min.	Max.	Mid.	25%	75%	Min.	Max.
6	128	−0.86	−1.45	0.00	−4.49	2.28	−0.89	−1.41	0.00	−4.64	11.11
7	245	−1.06	−1.69	−0.46	−6.14	5.42	−1.13	−1.74	−0.50	−6.14	4.15
8	464	−1.01	−1.51	−0.45	−5.25	12.18	−0.95	−1.51	−0.41	−5.25	13.38
9	659	−0.92	−1.43	−0.38	−5.01	4.99	−0.83	−1.32	−0.32	−5.41	5.45
10	783	−0.80	−1.29	−0.21	−6.67	9.55	−0.77	−1.23	−0.23	−7.85	9.55
11	957	−0.77	−1.29	0.00	−10.74	13.08	−0.77	−1.26	0.00	−11.63	15.84
12	1,005	−0.73	−1.22	0.00	−13.32	14.11	−0.68	−1.27	0.00	−11.35	12.57
13	1,019	−0.59	−1.13	0.00	−15.21	11.69	−0.60	−1.09	0.00	−14.35	11.37
14	950	−0.49	−1.03	0.00	−10.93	13.79	−0.50	−1.00	0.00	−10.02	12.57
15	951	−0.30	−0.90	0.00	−9.78	15.87	−0.32	−0.93	0.00	−10.45	15.87
16	877	−0.20	−0.79	0.00	−15.75	10.83	−0.15	−0.75	0.00	−15.75	11.11
17	1,627	−0.23	−0.66	0.00	−9.42	8.48	−0.22	−0.64	0.00	−9.13	8.88
18	3,305	0.00	−0.42	0.00	−17.17	13.95	0.00	−0.41	0.00	−13.74	12.82
19	3,050	0.00	−0.30	0.00	−14.47	18.34	0.00	−0.30	0.00	−14.47	18.56
20	3,244	0.00	−0.27	0.00	−10.80	16.19	0.00	−0.26	0.00	−10.80	17.14
21	3,449	0.00	−0.24	0.00	−13.73	17.00	0.00	−0.24	0.00	−13.97	14.91
22	3,638	0.00	−0.24	0.00	−14.97	17.36	0.00	−0.23	0.00	−16.66	17.36
23	3,729	0.00	−0.19	0.00	−12.10	15.54	0.00	−0.18	0.00	−13.61	15.44
24	3,804	0.00	−0.14	0.00	−17.26	20.44	0.00	−0.14	0.00	−16.55	20.94
25	3,636	0.00	−0.16	0.00	−15.72	18.43	0.00	−0.15	0.00	−13.97	17.11
26	3,546	0.00	−0.11	0.00	−16.20	18.99	0.00	−0.08	0.00	−16.84	17.68
27	3,573	0.00	0.00	0.00	−16.28	14.53	0.00	0.00	0.00	−18.25	15.28
28	3,376	0.00	0.00	0.00	−18.58	18.65	0.00	0.00	0.00	−16.93	18.88
29	3,196	0.00	0.00	0.00	−17.31	13.83	0.00	0.00	0.00	−18.48	14.04
30	3,127	0.00	0.00	0.00	−18.44	18.25	0.00	0.00	0.00	−17.47	17.11
31	2,982	0.00	0.00	0.00	−16.95	15.66	0.00	0.00	0.00	−17.38	15.66
32	2,892	0.00	0.00	0.00	−13.73	13.16	0.00	0.00	0.00	−13.73	16.28
33	2,911	0.00	0.00	0.00	−15.28	14.51	0.00	0.00	0.00	−12.32	15.14
34	2,727	0.00	0.00	0.00	−15.92	13.34	0.00	0.00	0.00	−14.95	12.63
35	2,817	0.00	0.00	0.00	−11.59	15.70	0.00	0.00	0.00	−13.46	14.72

Female											
Age	n	Right Eye					Left Eye				
		Mid.	25%	75%	Min.	Max.	Mid.	25%	75%	Min.	Max.
6	82	−0.90	−1.70	0.00	−8.93	2.04	−0.85	−1.64	0.00	−8.45	5.32
7	249	−1.06	−1.56	−0.50	−6.13	3.11	−1.04	−1.54	−0.67	−7.35	3.60
8	513	−1.00	−1.51	−0.55	−5.80	7.02	−1.00	−1.49	−0.50	−8.00	7.02
9	832	−1.00	−1.45	−0.56	−5.47	5.60	−0.96	−1.47	−0.50	−5.70	5.81
10	1,051	−0.89	−1.35	−0.40	−7.02	5.34	−0.87	−1.34	−0.41	−8.78	7.06
11	1,235	−0.76	−1.21	−0.30	−12.97	12.57	−0.77	−1.22	−0.27	−13.64	11.17
12	1,277	−0.74	−1.20	0.00	−8.90	9.51	−0.72	−1.20	0.00	−9.71	9.45
13	1,121	−0.67	−1.19	−0.17	−10.68	9.83	−0.62	−1.08	0.00	−10.20	11.83
14	1,040	−0.61	−1.02	0.00	−10.28	14.71	−0.58	−1.05	0.00	−9.94	14.84
15	855	−0.40	−0.91	0.00	−11.69	14.12	−0.36	−0.88	0.00	−10.09	14.28
16	757	−0.36	−0.81	0.00	−16.27	8.69	−0.32	−0.81	0.00	−16.27	8.69
17	1,290	−0.27	−0.67	0.00	−10.28	17.29	−0.27	−0.63	0.00	−8.97	16.33
18	2,682	−0.14	−0.49	0.00	−11.41	15.06	−0.10	−0.49	0.00	−12.06	14.21
19	2,890	0.00	−0.44	0.00	−15.47	15.29	0.00	−0.44	0.00	−15.00	16.21
20	3,475	0.00	−0.41	0.00	−19.13	15.12	0.00	−0.41	0.00	−21.59	14.87
21	3,865	0.00	−0.40	0.00	−15.97	16.75	0.00	−0.39	0.00	−15.83	15.58
22	4,350	0.00	−0.38	0.00	−22.20	24.53	0.00	−0.37	0.00	−19.03	24.53
23	4,402	0.00	−0.35	0.00	−21.16	19.46	0.00	−0.35	0.00	−20.72	17.66
24	3,940	0.00	−0.32	0.00	−12.77	18.65	0.00	−0.32	0.00	−14.01	18.20
25	3,597	0.00	−0.31	0.00	−18.29	14.13	0.00	−0.30	0.00	−18.25	14.85
26	3,387	0.00	−0.32	0.00	−19.55	20.88	0.00	−0.28	0.00	−20.64	20.88
27	3,276	0.00	−0.28	0.00	−15.67	18.55	0.00	−0.26	0.00	−14.57	16.95
28	2,980	0.00	−0.21	0.00	−20.38	20.28	0.00	−0.21	0.00	−19.05	18.59
29	2,730	0.00	−0.17	0.00	−20.55	21.86	0.00	−0.17	0.00	−21.23	20.91
30	2,535	0.00	−0.18	0.00	−13.93	17.66	0.00	−0.17	0.00	−14.16	16.91
31	2,286	0.00	−0.09	0.00	−14.47	18.55	0.00	−0.10	0.00	−14.23	17.77
32	2,239	0.00	−0.12	0.00	−16.28	20.22	0.00	−0.10	0.00	−16.63	18.74
33	2,106	0.00	0.00	0.00	−13.59	14.33	0.00	0.00	0.00	−13.59	14.11
34	1,891	0.00	0.00	0.00	−14.07	21.65	0.00	0.00	0.00	−13.30	21.65
35	1,883	0.00	0.00	0.00	−15.59	17.23	0.00	0.00	0.00	−15.75	16.77

This table provides the precise values (median, 25th percentile, and 75th percentile) of the annualized myopia progression rate (diopters/year) for each age group at the initial visit, stratified by sex and right/left eye, corresponding to the data visualized in Figure 4.

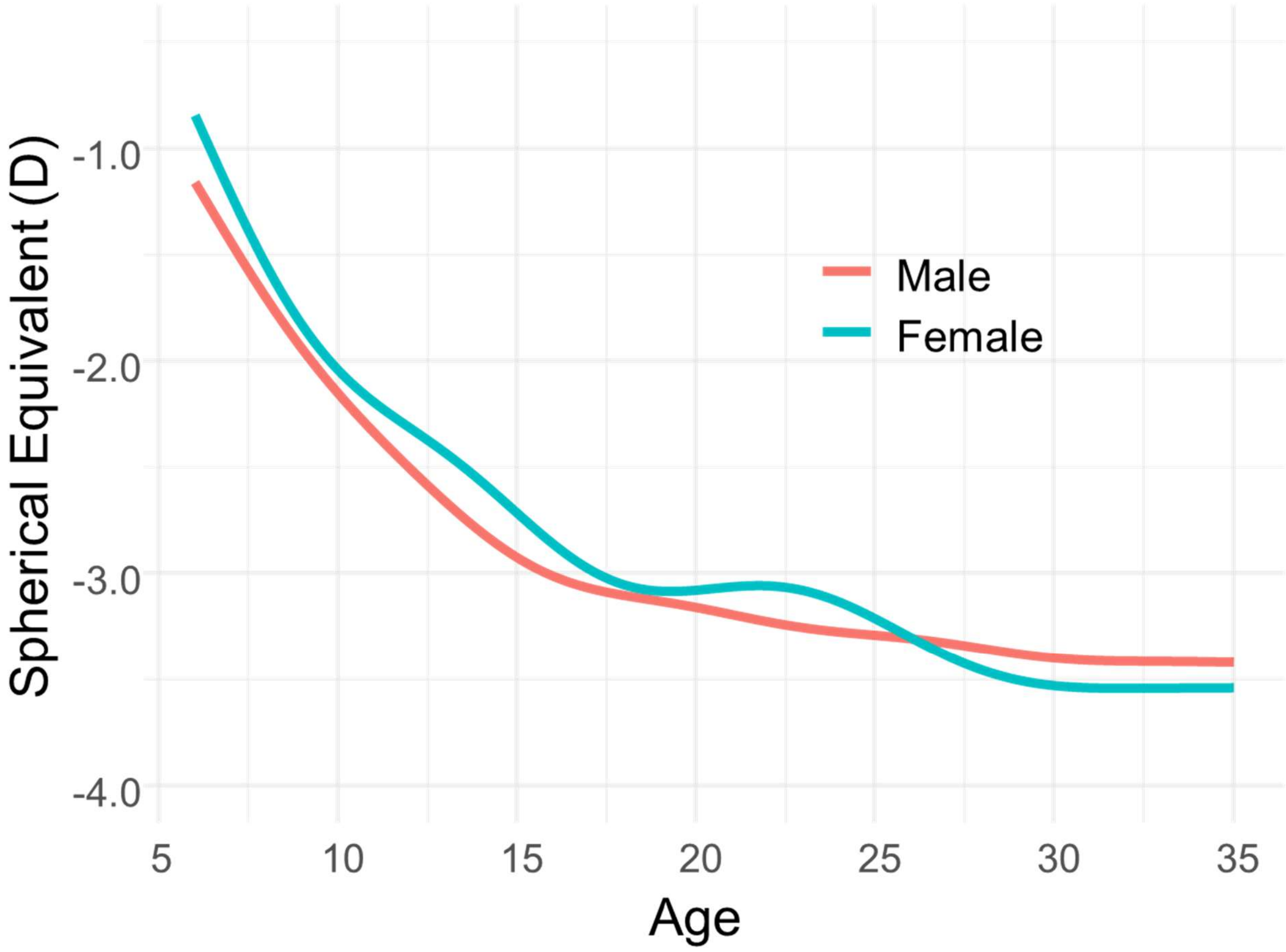

Supplementary Figure S1. Smoothed curves of age-related changes in spherical equivalent by sex

Generalized Additive Mixed Model analysis illustrating the differing trajectories of spherical equivalent (D) between men and women from ages 6 to 35 years.

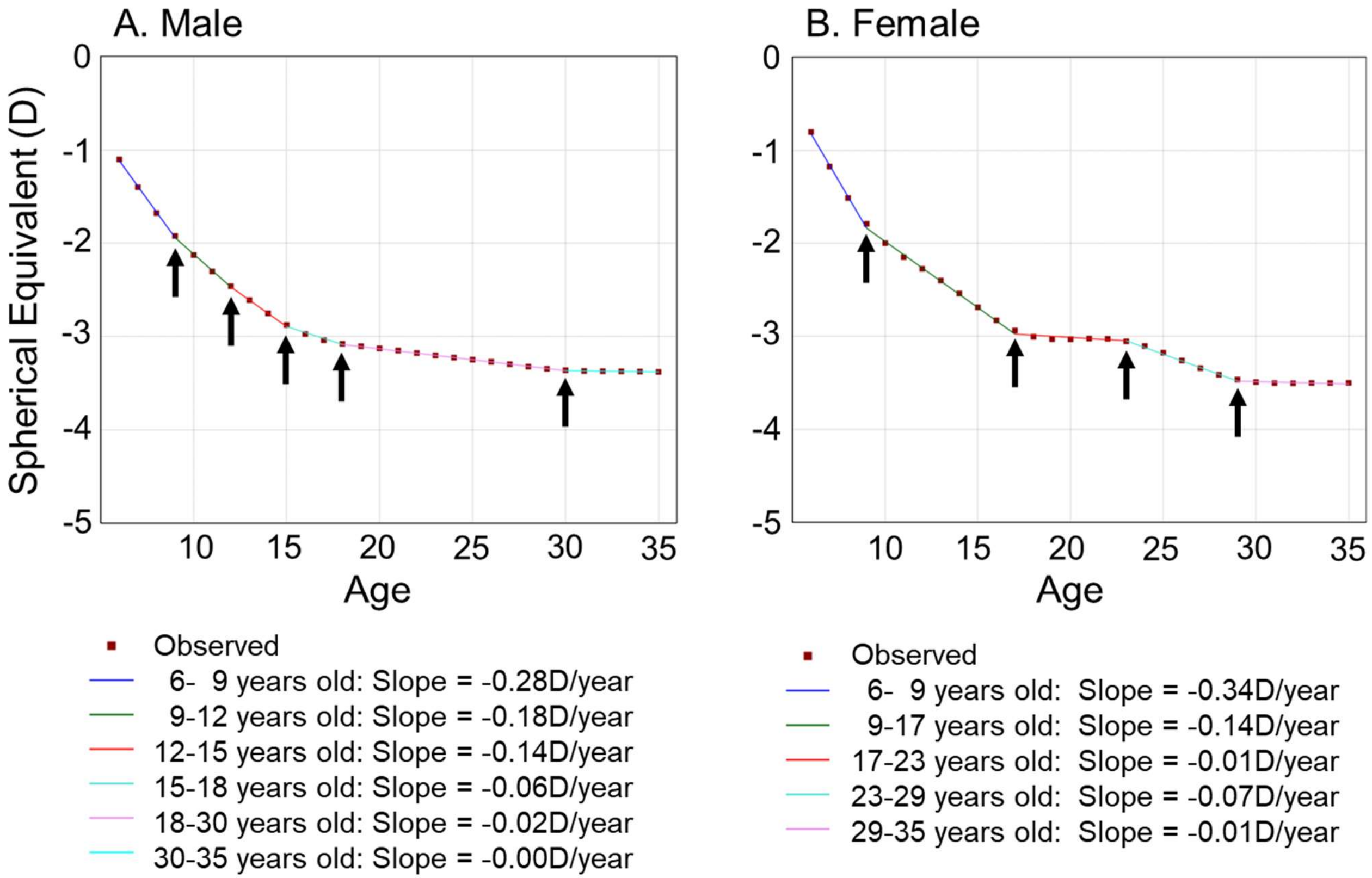

Supplementary Figure S2. Joinpoint regression analysis of spherical equivalent by age.

The models demonstrate the stabilization of spherical equivalent (D) in (A) men (five joinpoints) and (B) women (four joinpoints).

Asterisks indicate slopes significantly different from zero at the alpha = 0.05 level.

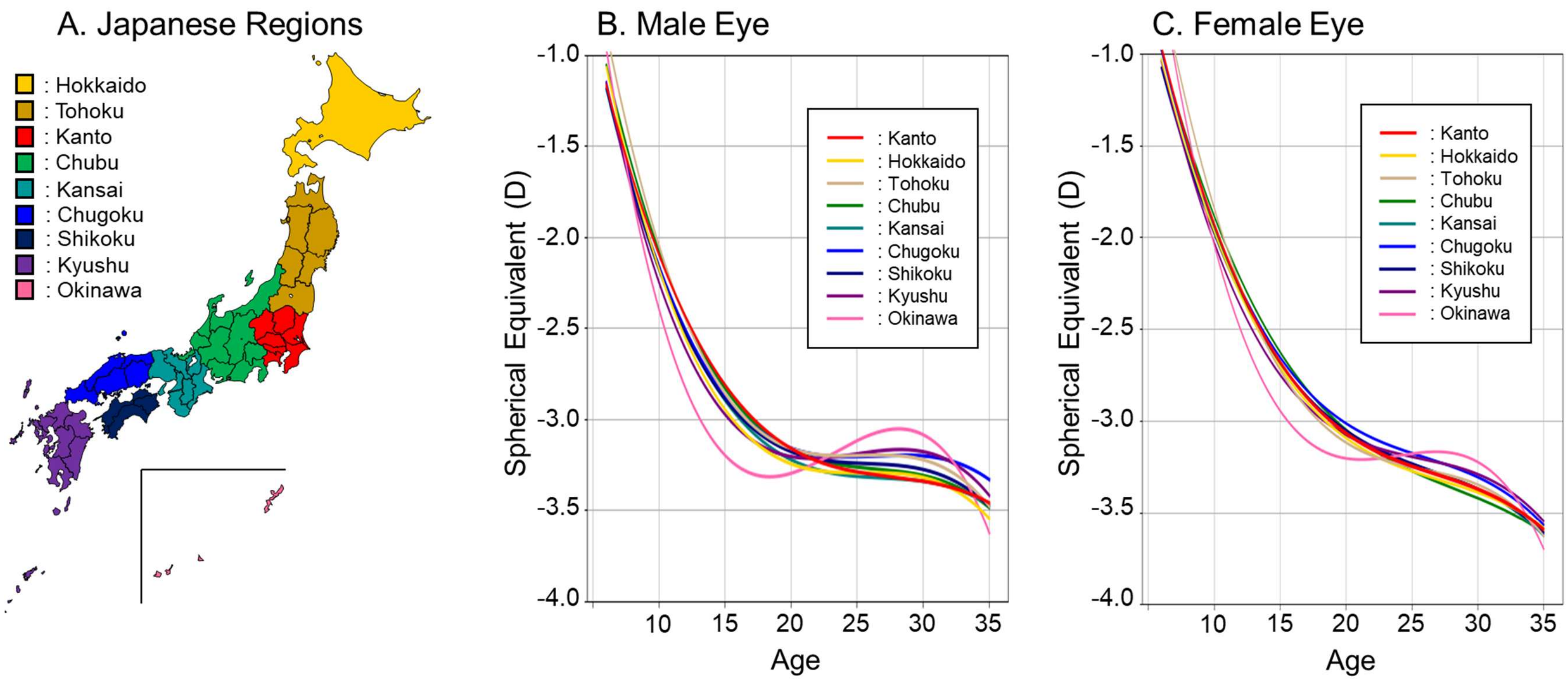

Supplementary Figure S3. Regional trends in spherical equivalent

(A) Geographic map illustrating the nine standard Japanese regions. (B, C) Generalized Additive Mixed Model smoothed curves comparing age-related refractive changes (spherical equivalent, D) across regions for male and female eyes, respectively.

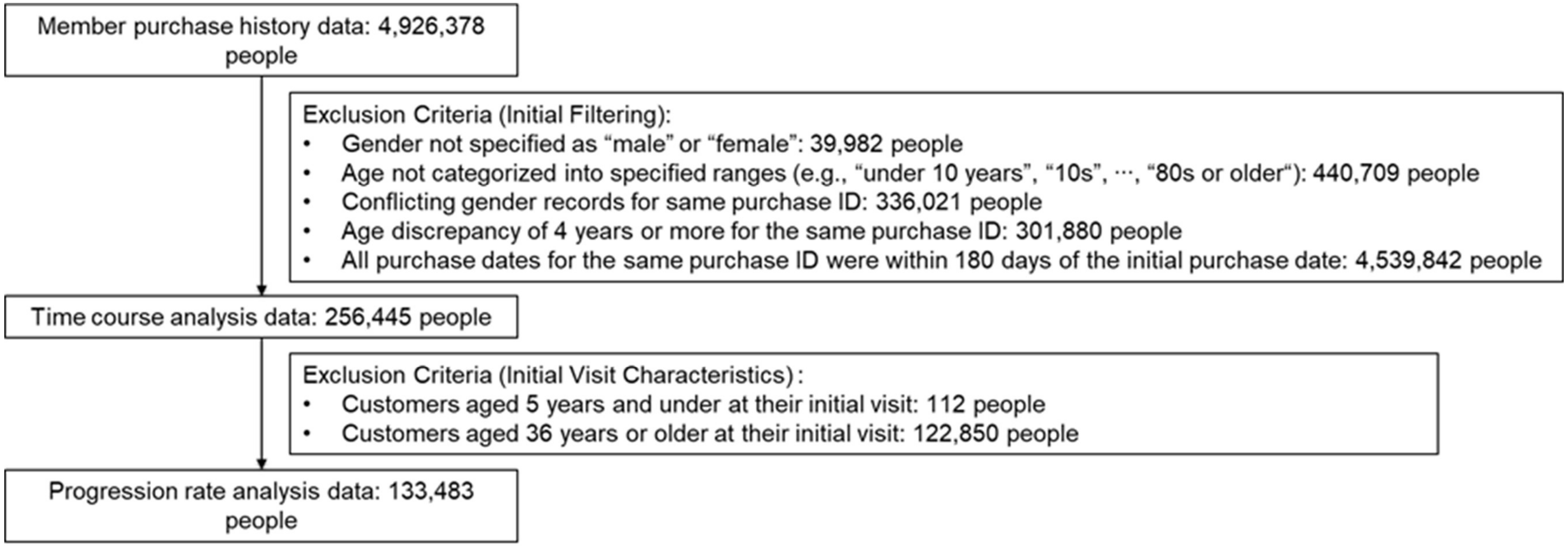

Supplementary Figure S4. Flowchart of study participants included in the longitudinal analysis

This diagram outlines the multi-step exclusion criteria (initial filtering and baseline visit characteristics) applied to 4,926,378 customers' purchase history data, resulting in a final cohort of 133,483 individuals for the progression rate analysis.

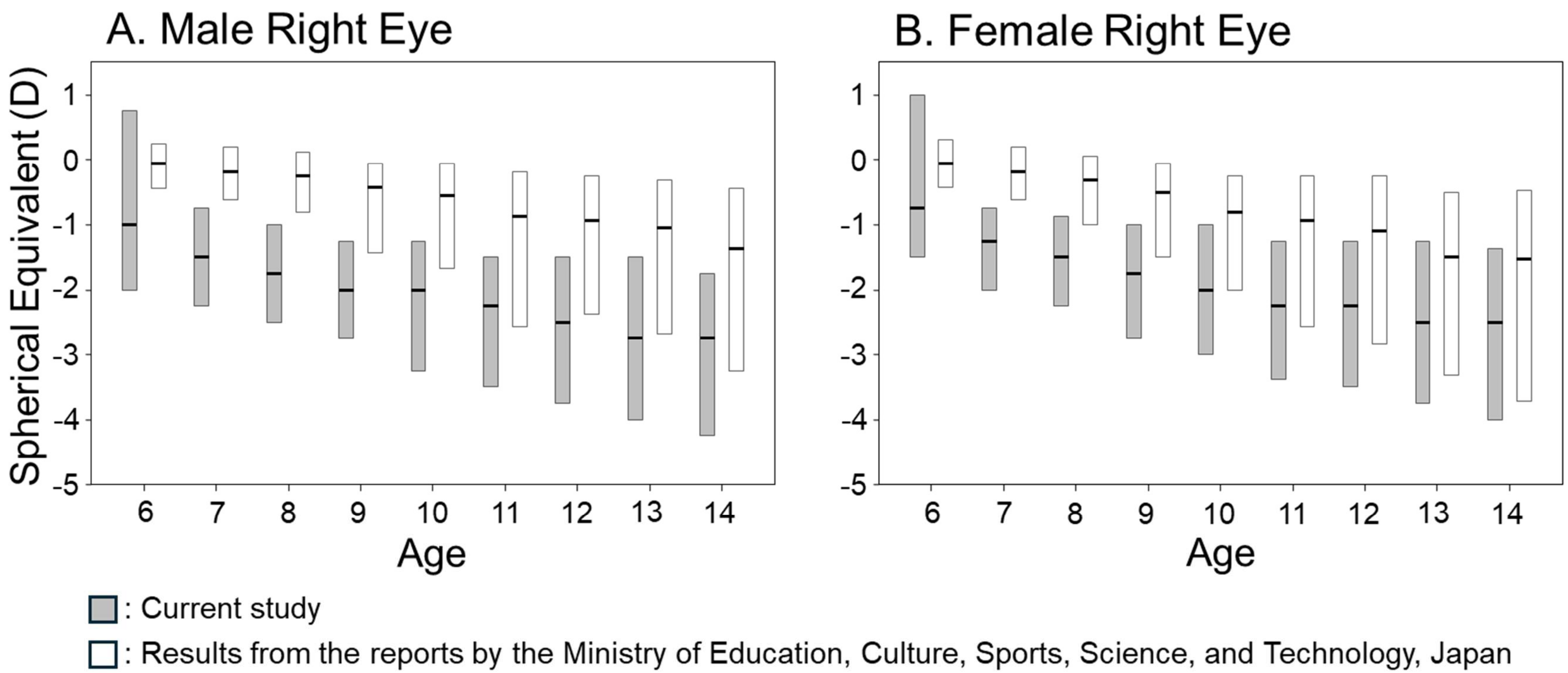

Supplementary Figure S5. Comparison of spherical equivalent with national school survey data

Box plots comparing the spherical equivalent (D) by age between the current study and the reports from the Ministry of Education, Culture, Sports, Science, and Technology, Japan, for (A) male right eye and (B) female right eye from ages 6 to 14 years.
